# Dynamic tensile properties, deformation, and failure testing of impact-loaded coal samples with various water content

**DOI:** 10.1038/s41598-021-86610-7

**Published:** 2021-03-29

**Authors:** Zhen Wei, Ke Yang, Xiao-Lou Chi, Xiang He, Xin-Yuan Zhao, Ji-qiang Zhang

**Affiliations:** 1 State Key Laboratory of Mining Response and Disaster Prevention and Control in Deep Coal Mines, Anhui University of Science and Technology, Huainan, 232001 China; 2 Institute of Energy, Hefei Comprehensive National Science Center, Hefei, 230031 China; 3 Key Laboratory of Mining Coal Safety and Construction Efficiency of Anhui Province and Ministry of Education, Anhui University of Science and Technology, Huainan, 232001 China; 4 China University of Mining & Technology (Beijing), Beijing, 100083 China

**Keywords:** Civil engineering, Energy infrastructure, Energy storage, Mineralogy

## Abstract

Disc coal samples with different water content were tested using the split Hopkinson press bar test system. Their dynamic tensile failure process was monitored via an ultra-high-speed digital image correlation system. The deformation trend and failure characteristics as a function of the water content were analyzed, and the water content effect on dynamic mechanical properties was investigated. The results demonstrated that the dynamic stress–strain curve of the coal samples consisted of four stages. As the water content increased, the coal sample brittleness degraded, while its ductility was enhanced. Quadratic polynomial functions can describe dynamic peak stress, peak strain, and loading pressure. Under different loading pressures, the dynamic peak stress exhibited a concave bending trend as the water content increased. The coal sample's dynamic tensile strength had a strong rate correlation, and the saturated coal sample exhibited the highest rate correlation. Under high-rate loading, the inertia effect and the Stefan effect of water in coal samples hinder the initiation and propagation of coal sample cracks, improving the coal sample's strength. The research results provide a basic theoretical basis for the prevention and control of rock burst in coal mines.

## Introduction

Water is one of the main factors affecting the mechanical properties of materials. In coal mining engineering, high ground pressure disturbances are prone to inducing secondary engineering disasters, producing water-conducting cracks, thus forming a large amount of mine water^1,2^. The construction of pumped storage power generation projects in underground reservoirs can effectively reduce the water loss caused by mine water discharge, improving the power system’s feasibility and safety^3–5^. The preset coal pillars and artificial coal mining walls constitute the underground reservoir and soaked by groundwater for a long time^6^. Therefore, it is necessary to consider the interaction between water and coal during coal mining, the design of water-proof pillars in aquifers, and the excavation and support of water-rich roadways. The coal rock structure is complex and changeable; thus, the mechanical performances under impact loads are quite different from those under static ones^7,8^. As the underground coal pillar wall of the pumped storage power generation project is subjected to long-term dynamic load, to prevent coal rock damage caused by dynamic load impact from causing catastrophic accidents, it is necessary to understand the impact failure characteristics of coal samples with different water content^9^. Hence, in designing underground reservoir coal pillars and roadway safety support, it is crucial to reveal the impact failure mechanism of coal rock via the dynamic tensile test of coal samples with different water content.

Many studies on the mechanical performance of water-soaked coal have been carried out in recent years. Still, most of them were focused on the impact of water on coal sample mechanical performance under static load, which revealed the static tensile performances of saturated coal samples. The results show that static loads have a weakening effect on coal samples' mechanical performances^10,11^. Various field engineering cases have shown that dynamic instability and failure often occurs in coal rock^12^, while the dynamic tensile performance of coal rock has important mechanical features, which is of great significance for the control of coal rock stability under impact load and the study of disaster mechanism caused by rockburst. At present, scholars have carried out extensive research on the mechanical performances of coal rock under dynamic load with the separated Hopkinson bar (SHPB) test device, and some significant research results have been achieved. Li et al*.*^13^ performed the granite layer fracture test using the improved Hopkinson bar device and analyzed the failure characteristics of hard rock fracture under impact loads. Yuan et al*.*^14^ carried out impact compression tests on sandstones with different water content using an SHPB device. The variation trend of sandstone dynamic uniaxial compressive strength with water content was obtained. Kim et al*.*^15^ studied the effect of water content and loading rate on sandstone's physical performances. Zhao et al*.*^16^ investigated the effects of bedding angle and loading rate on the initiation and propagation of cracks in coal samples. Exploring the dynamic deformation and failure characteristics of layered sandstone, Yang et al.^17^ carried out the dynamic Brazilian disk test of sandstone at different bedding angles combined with the digital image correlation (DIC) method. Ai et al*.*^18^ carried out a dynamic sandstone bar test using a separated SHPB. They analyzed the relationship between crack propagation and dynamic mechanical performance under impact load combined with DIC technology. Insofar as energy is the inherent factor during the material failure process, which changes throughout the entire process of material deformation and fracture, it is the main way to study the failure mechanism of coal rock by analyzing energy dissipation^19–21^. Based on the principle of energy dissipation, scholars have also carried out a series of experimental studies on the dynamic deformation and failure of different coal rock and analyzed the energy dissipation^22^, fractal characteristics^23^, and energy storage characteristics^24^ of coal rock, thus improving comprehension of energy dissipation in the process of coal rock fracture. However, the dynamic tensile test results on coal samples are different, as the strata coal samples contain a certain amount of moisture. Therefore, it is necessary to study the dynamic tensile characteristics and evolution of coal samples with different water contents.

The Brazilian disc indirect tensile test is the simplest and most effective method to test the tensile performances of coal rock. Based on the static tensile test principle and after long-term theoretical substantiation and device innovation efforts, an SHPB test device was developed to realize the dynamic tensile performance test of materials^25^. To further study the deformation and failure features of coal samples with different water contents under impact load, the hard coal from the Hujiahe coal mine in the Binchang mining area, Shaanxi was selected. The SHPB test system of the Anhui University of Science and Technology with the pressure rod diameter of 50 mm was used to deeply analyze the deformation and failure features of coal samples with different water contents under various loading pressures. During the test, a high-speed digital camera was used to capture and record the crack propagation process of coal samples. Thus, the strain field of coal samples under different loading pressures, the dynamic evolution process of the displacement field, crack propagation behavior, and mechanical performance trends were analyzed, revealing the deformation and failure mechanism of coal samples with different water contents under high loading rates.

## Experimental

### Sample preparation

Coal samples applied in the test were taken from the 401,103 workface of the Hujiahe coal mine in the Binchang mining area. According to the international recommendations for the Brazilian split test of rocks to determine the dynamic tensile strength of materials^26^, the coal block with good integrity and homogeneity was cored and cut into Φ50 mm × 25 mm disc samples. To meet the SHPB test requirements and reduce the effect of end-face friction, the SHM-200 double-end face grinder was used to grind and polish the disc samples. The non-parallelism of the end face in the coal sample should be less than 0.05 mm, while its diameter’s deviation should be less than 0.02 mm, the processed sample ensures that there is no obvious crack on the surface. To ensure the correlation of the samples' physical and mechanical performance and avoid differences in the samples' structure and composition, all samples were taken from the same coal block.

**Figure 1:**
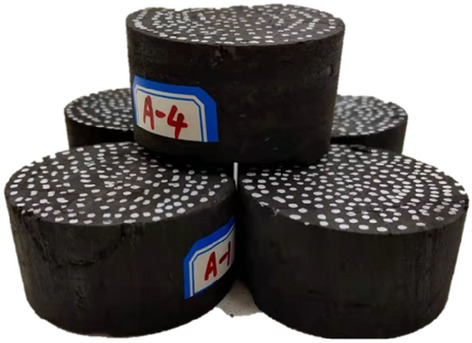
Typical coal sample.

Typical coal samples are shown in Fig. 1.

The experimental objects were subdivided into three groups, 15 samples in each group, and the experimental coal samples were prepared. The specific steps were as follows:

1. All coal samples were placed in a constant temperature drying oven to obtain dry coal samples (w = 0), the temperature was 20℃, the drying time was 48 h, and their weight was $m_{dry}$.
2. According to GB/T23561.5–2009, saturated coal samples were prepared using a vacuum suction device, which weight was recorded as $m_{sat}$. The saturated water content of the coal sample can be calculated as in^27^:
  1 $$ w_{sat} = \frac{{m_{sat} - m_{dry} }}{{m_{dry} }} \times 100 $$

This test involved dry, natural, and saturated coal samples, each group consisting of 15 replicates. Table 1 shows the prepared coal samples' properties, with the average water content of saturated coal samples of 3.174%.

**Table 1:** The water content in coal samples.

Sample	Water content *w*/%			Average water content/%
BD-1-0.30	0	0	0	0 (Dry samples)
BD-1-0.35	0	0	0	
BD-1-0.40	0	0	0	
BD-1-0.45	0	0	0	
BD-1-0.50	0	0	0	
BD-2-0.30	2.137	2.130	2.166	2.136 (Natural samples)
BD-2-0.35	2.128	2.140	2.173	
BD-2-0.40	2.108	2.119	2.156	
BD-2-0.45	2.148	2.103	2.113	
BD-2-0.50	2.157	2.152	2.143	
BD-3-0.30	3.140	3.158	3.165	3.174 (Saturated samples)
BD-3-0.35	3.192	3.154	3.132	
BD-3-0.40	3.134	3.157	3.128	
BD-3-0.45	3.159	3.143	3.183	
BD-3-0.50	3.170	3.177	3.196	

Before the test, spots with size, density, and irregularity characteristics, which met the DIC method's calculation requirements, were painted on the surface of coal samples by spray painting^28^. During the test, the spots of digital images on the surface of coal samples were tracked to obtain the surface strain information in the dynamic tensile process of coal samples^29^.

### Test system and scheme

**Figure 2:**
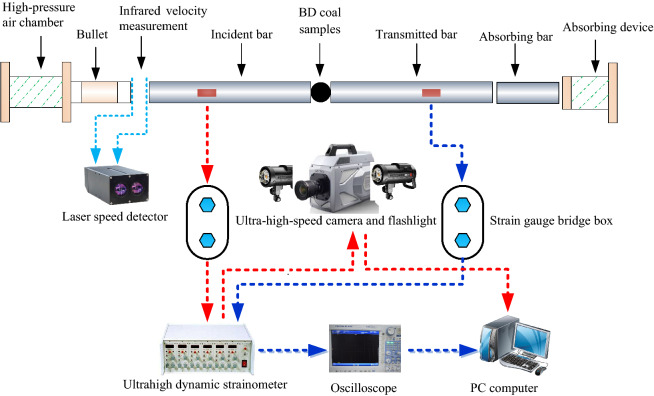
SHPB and high-speed DIC test system.

The SHPB test system with an opposite-type punch developed by the Central South University, China, was applied in this experiment, as shown in Fig. 2. The test device's pressure rod (of 50 mm in diameter) and punch were made of high-strength alloy steel with a density of 7800 kg/m^3^ and an elastic modulus of 210 GPa. The impact waveform was a sine wave with a longitudinal wave velocity of 5190 m/s^30^. During the test, the opposite punch hit the input rod at a certain speed in the axial direction, generating a stress wave in the input rod. When the stress wave was transmitted to the coal sample, a reflected stress wave was transmitted to the input rod. Meantime, the coal sample transmitted a stress wave to the output rod. Then, the strain gauge pasted on the pressure rod received the corresponding stress wave signal^31^.

The test data acquisition equipment included an SDY2107A super dynamic strain gauge, Yokowaga-DL850E oscilloscope, and ultra-high-speed DIC test system. The above system comprised a FASTCAM SA-Z high-speed camera, lighting flash, trigger, and synchronous control system. Before the test, the high-speed camera's shooting speed was set at 120,000 fps, and the image resolution was 256 pixels × 408 pixels, which could capture the deformation and failure features of the coal samples and meet the test requirements. The velocity and incident amplitude of opposite-type punch were controlled by adjusting nitrogen pressure.

A spare coal sample was tested before the experiment to determine a suitable loading air pressure.

The results obtained show that the tensile strength of the coal samples was relatively small. Hence, the loading pressures of 0.30, 0.35, 0.40, 0.45, and 0.50 MPa were applied in the test. Three specimens were tested at each of the above loading pressures, and a total of 45 dynamic tensile tests were carried out. After the test, the effective results that met the standard requirements were selected for comparative analysis.

During the test, to improve the test results' accuracy, the vaseline was applied to the contact zone between the pressure rod and the coal sample, thus reducing the friction coefficient and friction effect.

## Results and analysis

### Verification of the dynamic stress equilibrium

**Figure 3:**
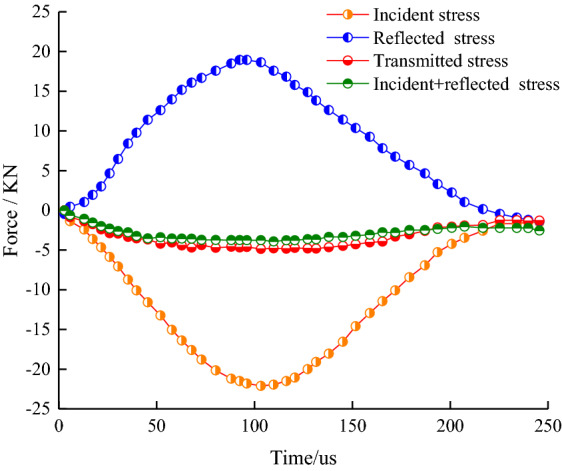
Dynamic stress equilibrium of typical samples.

In the abbreviations of samples listed in Table 1, “BD” in BD-1-0.30 means the Brazilian splitting test, while “1” represents the first group of tests, and “0.30” stands for the loading pressure value. For the SHPB test, the stress equilibrium before samples' failure is the prerequisite for the effective dynamic test results. During impact loading, the strain gauge on the incident rod and transmission rod measured electrical signals. Through signal transformation, the change of strain (stress) in the elastic rod at both ends of the sample with time can be obtained. As shown in Fig. 3, the typical sample's stress equilibrium curves in a dynamic tensile test were drawn according to the test results. As observed, the transmitted wave curve, the incident and reflected waves' superimposition curve overlap during the impact loading process, indicating that the stress at both ends of the sample is balanced before the sample's failure, and the test results are valid. During the data processing, all test results were strictly verified by stress equilibrium, and the test results that did not meet the standards were eliminated.

### Dynamic stress–strain curves

Under impact loading, the voltage signals on the incident rod and the transmission rod were collected by ultra-dynamic strain gauge and converted into strains based on the 1D stress wave theory. Then, combining Eqs. (2)–(4), the coal sample's stress $\sigma (t)$, strain $\varepsilon (t)$, and strain rate $\mathop \varepsilon \limits^{ \cdot } (t)$ under impact load were calculated with a three-wave method to obtain the dynamic tensile stress–strain curve of the coal sample.

2 $$ \sigma (t) = \frac{EA}{{2A_{s} }}\left[ {\varepsilon_{I} \left( t \right) + \varepsilon_{R} \left( t \right) + \varepsilon_{T} \left( t \right)} \right] $$

3 $$ \varepsilon (t) = \frac{c}{{l_{s} }}\int_{0}^{t} {\left[ {\varepsilon_{I} \left( t \right) - \varepsilon_{R} \left( t \right) - \varepsilon_{T} \left( t \right)} \right]} dt $$

4 $$ \mathop \varepsilon \limits^{ \cdot } (t) = \frac{c}{{l_{s} }}\left[ {\varepsilon_{I} \left( t \right) - \varepsilon_{R} \left( t \right) - \varepsilon_{T} \left( t \right)} \right] $$

where c, E, and A are the elastic wave velocity, elastic modulus, and cross-sectional area of the compression rod, respectively; As and $l_{s}$ are the cross-sectional area and length of the coal sample, while $\varepsilon_{I} \left( t \right)$, $\varepsilon_{R} \left( t \right)$ and $\varepsilon_{T} \left( t \right)$ are the incident strain, reflected strain, and transmitted strain, respectively.

**Figure 4:**
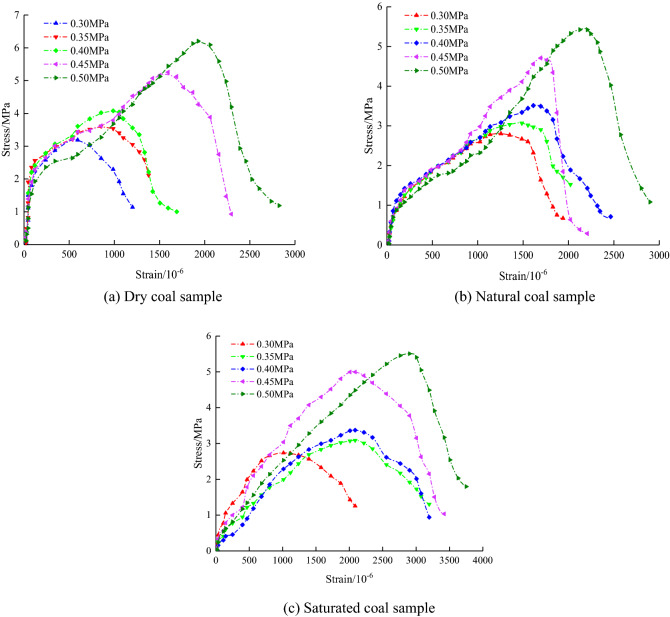
Dynamic stress–strain curves of coal samples under different loading pressures.

Figure 4 shows the dynamic tensile stress–strain curve of typical coal samples under impact load. As observed, a coal sample's stress–strain curve under dynamic load can be roughly subdivided into four stages^31,32^. Stage I is the compaction stage. The coal sample's internal microcracks are closed under the action of external force, showing the trend of rapid first and then gradual slowing down, and the $\sigma - \varepsilon$ curve shows a concave bending trend. However, the loading rate of impact load is much higher than that of static or quasi-static load, resulting in the closure of internal microcracks; thus, the curves' feature is not obvious. The second stage is the elastic (linear) deformation part, which is mainly due to the enormous elastic energy stored inside the coal sample under external load. The curve approaches a straight line, which slope is equal to the coal sample dynamic elastic modulus. The third stage is the plastic deformation stage, which is mainly due to the plastic deformation of the coal sample under external force. The $\sigma - \varepsilon$ curve is concave, and its slope is gradually reduced. The latter value varies with water content and reaches zero in the peak stress position. The fourth stage is the failure stage, where the coal sample undergoes brittle (ductile) failure under external load. Thus, the curve reflects different states with a negative slope.

The results demonstrated that dynamic tensile performances of coal samples with various water contents are significantly different. Specifically, the greater the water content of the coal sample, the greater the peak strain. Besides, the elastic stage of the stress–strain curve is longer than the plastic stage. The overall tendency is the right shift, indicating reduced brittleness, enhanced ductility, and exacerbated coal samples’ damage^33^.

### Trend of the dynamic peak stress

**Figure 5:**
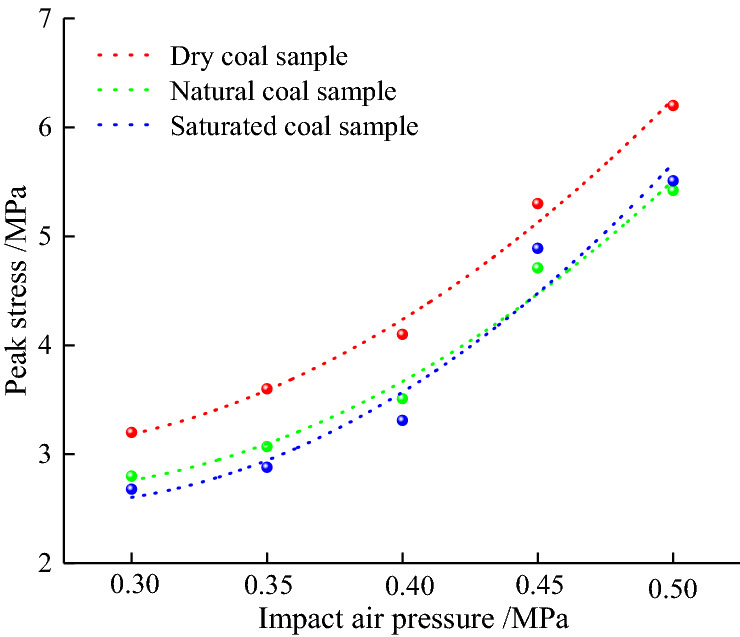
The trend of coal sample peak stress with loading pressure.

Figure 5 illustrates the trends of dynamic tensile peak stress and coal samples' loading pressure with different water contents. As observed, the coal sample's dynamic peak stress increased with the loading pressure, indicating an obvious rate effect. The dynamic peak stress (σd) and loading pressure (p) can be described by:

5 $$ \sigma_{d} = ap^{2} + bp + c $$

where a, b, and c are coefficients of the quadratic function.

The dynamic peak stress function coefficients of the coal sample are listed in Table 2.

**Table 2:** Peak stress and loading pressure fitting coefficient of different samples.

Water content	*a*	*b*	*c*	Correlation coefficient *R*^2^
Dry	0.4857	− 2.3457	5.84	0.9915
Natural	0.4685	− 2.3725	5.66	0.9816
Saturated	0.5685	− 3.0145	6.53	0.9576

As observed, the correlation coefficient (*R*^2^) of σd and *p* was reduced to 0.9576, indicating a good correlation between them, and the fitting coefficient a was greater than 0. Thus, the curve shape is concave, as shown in Fig. 5. As observed, the loading pressure and dynamic peak stress of dry samples (w = 0%) increased from 0.3 to 0.5 MPa and from 3.22 to 6.16 MPa, demonstrating a significant positive effect of the loading rate on the dynamic peak stress of dry coal sample. The results show that with the increase of loading rate, water has resistance to crack propagation and hinders its initiation and propagation, that is, water content has strengthening effect on dynamic tensile strength of coal samples, but the strengthening effect is less. At *p* = 0.4–0.5 MPa, the saturated coal sample's dynamic peak stress increased faster, indicating that under high loading pressure, the water content has a strengthening effect on dynamic tensile strength. If *p* = 0.3–0.5 MPa, peak stresses are 2.63, 2.78, 3.11, 4.48, and 5.36 MPa, respectively. Compared with the dry coal samples with the same loading pressure, the peak stresses were reduced by 32.7, 22.7, 24.1, 16.0, and 12.9%, indicating that the greater the water content under the same loading pressure, the lower the dynamic tensile strength of the coal sample.

**Figure 6:**
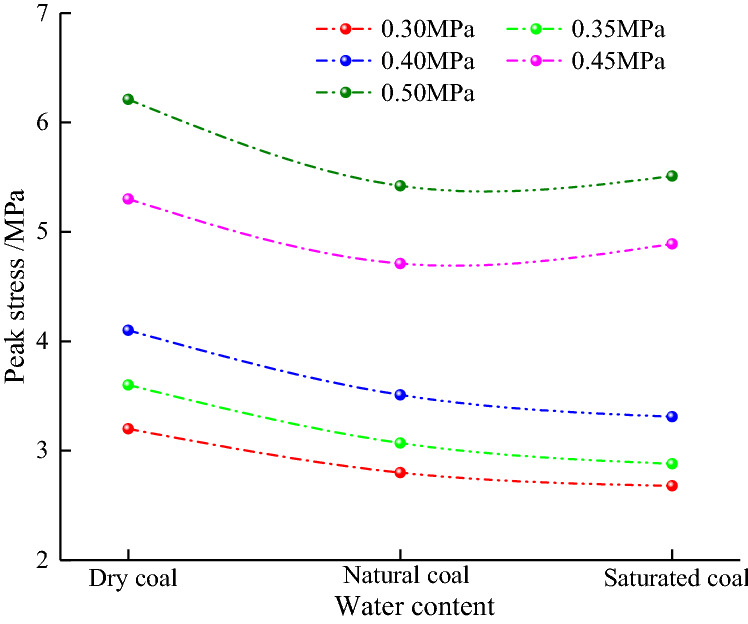
The trend of coal sample peak stress with water content.

Figure 6 shows the trend of dynamic tensile peak stress of coal samples as a function of water content at different loading pressures. As observed, the curve of dynamic peak stress of coal samples with water content shows a trend of asymmetric concave bending trend, and the peak stress curve shows an overall upward trend as the loading pressure increases. If *p* = 0.3–0.4 MPa, as the water content increased, the coal sample peak stress gradually decreased. If *p* = 0.4–0.5 MPa, the coal sample's peak stress decreased first and then increased with the water content, while the peak stress of natural samples always exceeded that of saturated ones. At the loading pressure of 0.50 MPa, the dynamic peak stresses of dry, natural, and saturated coal samples were 6.21, 5.42, and 5.51 MPa, respectively, indicating that the coal sample water content had a strong effect on its peak stress.

### Trend of dynamic peak strain

**Figure 7:**
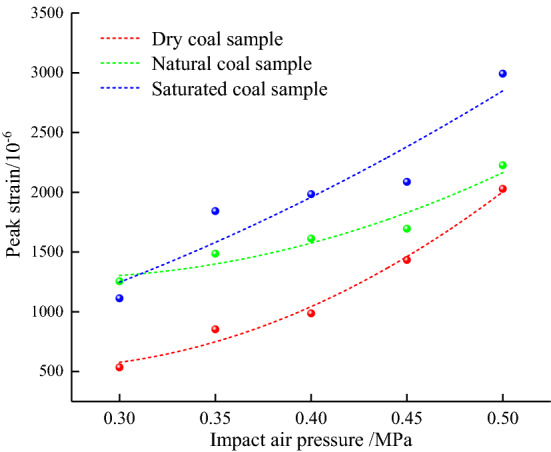
Peak strain versus loading pressure.

Figure 7 shows trends of dynamic peak strains of coal samples with different water contents as a loading pressure function. As observed, peak strains of coal samples with different water contents increased with loading pressure. The correlation of dynamic peak strain $\varepsilon_{d}$ and *p* can be described as follows:

6 $$ \varepsilon_{d} = dp^{2} + ep + f $$

where d, e, f are coefficients of the quadratic function.

Values of coefficients d, e, and f are listed in Table 3. As observed, the minimum correlation coefficient R2 between the dynamic peak strain of coal samples at different rates and the loading pressure was equal to 0.9358, indicating a good correlation. Moreover, the quadratic function coefficient d was higher than zero, and the curve shape was concave, as shown in Fig. 7. As observed, dry samples' loading pressure and dynamic peak strain increased from 0.30 to 0.50 MPa and by 279.06%, respectively, showing a strong loading rate effect. The peak strain curves of natural and saturated coal samples were located above that of dry coal samples. The peak strain curves of saturated coal samples were the highest. Thus, the peak strain of coal samples increased with water content. The loading pressure had a positive effect on the dynamic peak strain of coal samples.

**Table 3:** Fitting coefficients of peak strain versus loading pressure curves.

Water-content type	*d*	*e*	*f*	Correlation coefficient *R*^2^
Dry	24.714	− 12.637	2.144	0.9871
Natural	15.914	− 8.429	2.400	0.9358
Saturated	6.085	3.737	− 0.475	0.9674

**Figure 8:**
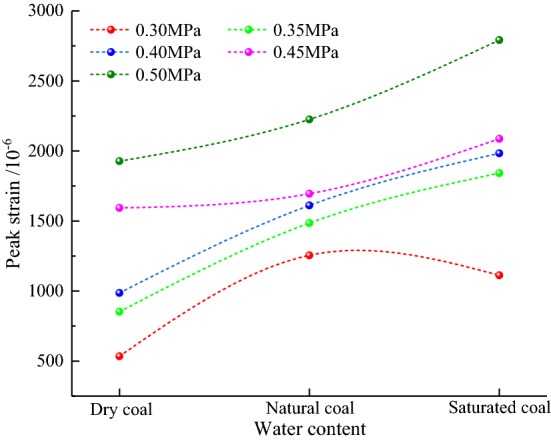
Peak strain versus water content.

Figure 8 shows trends of peak dynamic tensile strain of coal sample as a function of water content at different loading pressures. At loading pressure of 0.3 MPa, the peak strain first increased and then decreased as the water content increased; at loading pressure of 0.35 and 0.4 MPa, peak strain exhibited a concave growth as the water content increased; at loading pressure of 0.45 and 0.5 MPa, peak strain had a convex growth as the water content increased. With an increase in the loading pressure, peak strains of coal samples with different water contents shifted upwards at different rates.

With the increase of loading pressure, the dynamic tensile strength of coal samples increases gradually and has a good rate correlation. Water content will affect the post-peak deformation behavior of coal samples. After the dry samples reach the peak load, there will be obvious stress drop phenomenon, while after the water-containing samples reach the peak load, the stress does not fall immediately, but slowly decreases, showing the characteristics of plastic deformation.

The mechanical properties of water bearing coal samples are the result of the interaction between softening and strengthening of water. When the loading rate is low, water makes clay mineral particles expand, which leads to the disintegration of two adjacent particles and the increase of particle spacing, resulting in the decrease of liquid bridge force, the decrease of interfacial cohesion and the dominant role of water softening. When the loading rate is high, the inertial action and viscous stress are enhanced, and the enhancement of water is dominant. The strength of saturated coal sample may be greater than that of dry sample.

## Dynamic failure of coal samples

### Spatiotemporal evolution characteristics of the strain field

**Figure 9:**
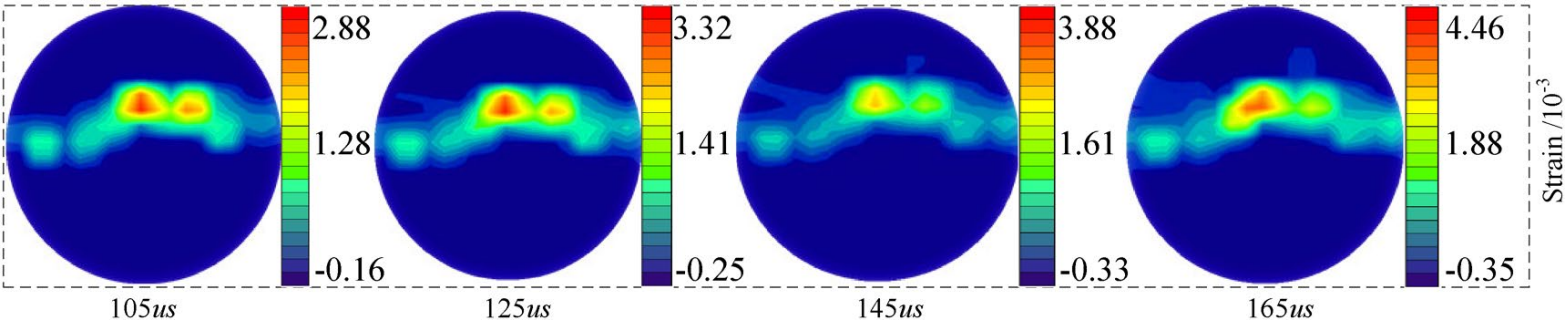
Dry coal sample maximum principal strain cloud diagram.

The coal sample's initial spot pattern under impact load was regarded as a reference. The spot pattern at typical moments in the loading process was selected as the deformation image and then analyzed with the PhotoInfor and PostViewer digital image processing software^34^. Figure 9 shows the maximum principal strain cloud diagram of the dry coal sample at the loading pressure of 0.30 MPa (tensile strain is positive and compressive strain is negative). As observed, the maximum principal strain concentration area is first formed in the middle of the sample under impact load and propagated rapidly, indicating that the disk sample at the center reaches the critical failure value at first, and then breaks and rapidly propagates to form macroscopic cracks parallel to the loading direction.

During the dynamic tensile test of the coal sample, the maximum principal strain field exhibited a good symmetry, that is, the sample was in the dynamic equilibrium state under impact loading. Under the radial symmetric impact load, the tensile strain at the coal sample center was always the maximum. The microcrack was generated at the sample center and split along the radial direction. This was consistent with the static-load Brazilian disk test results. At the same time, the tensile stress in the vertical loading direction was the main factor for the initiation and propagation of cracks.

**Figure 10:**
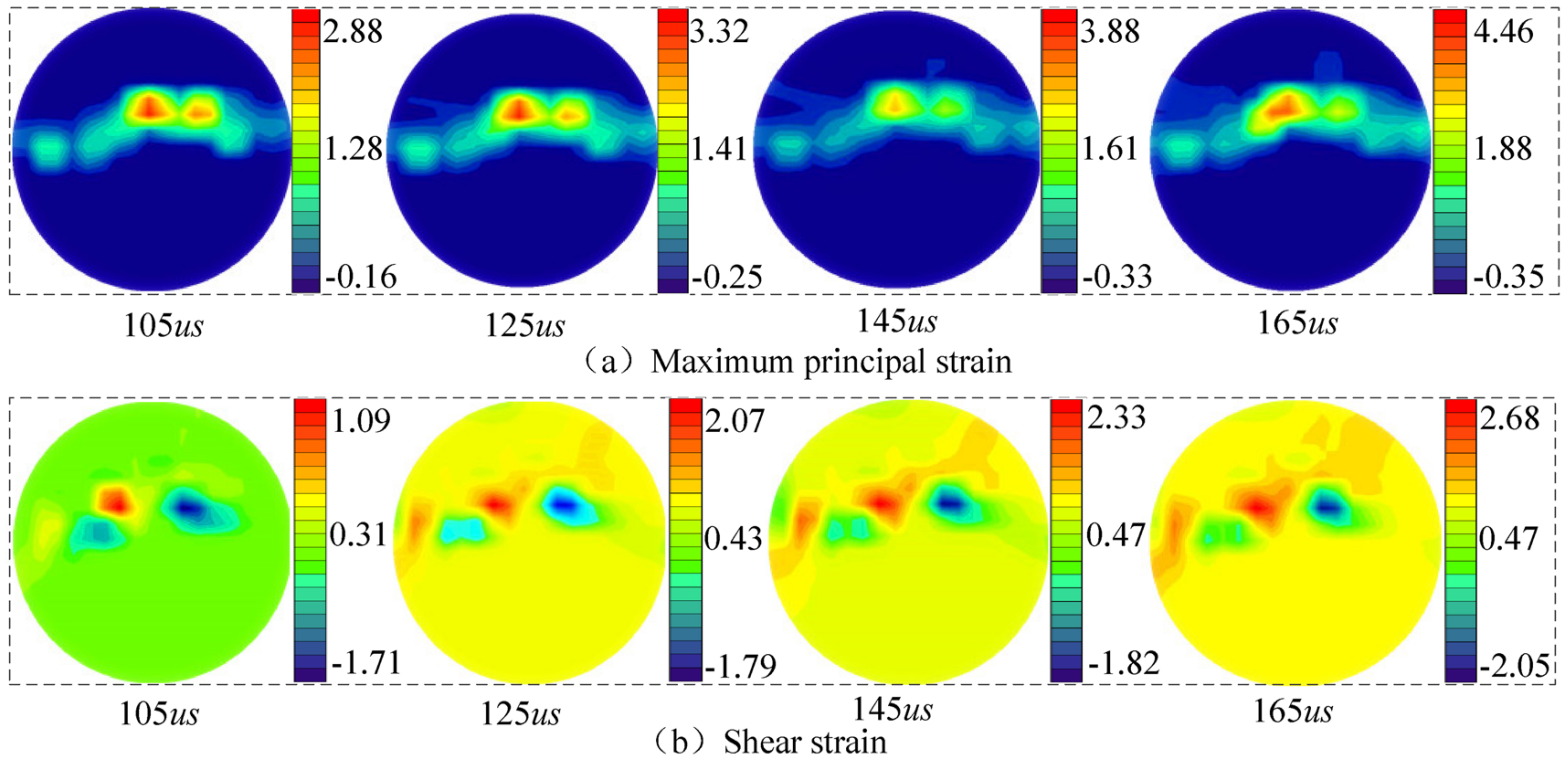
Strain field distribution in natural coal sample at loading pressure of 0.30 MPa.

Figure 10 shows the cloud diagram of the natural disk coal sample's strain field cloud map at an impact load of 0.30 MPa. As observed, a significant strain concentration occurs in the coal sample's central position under a natural state. The crack first initiates and propagates in the central area under the combined action of tensile and shear stresses. In contrast to dry coal samples, a wide-range strain concentration zone existed in the natural coal sample due to the water content effect. Combined with the shear strain cloud diagram, the shear strain value under impact loading is smaller, indicating that the coal sample's failure mode is an open fracture type.

**Figure 11:**
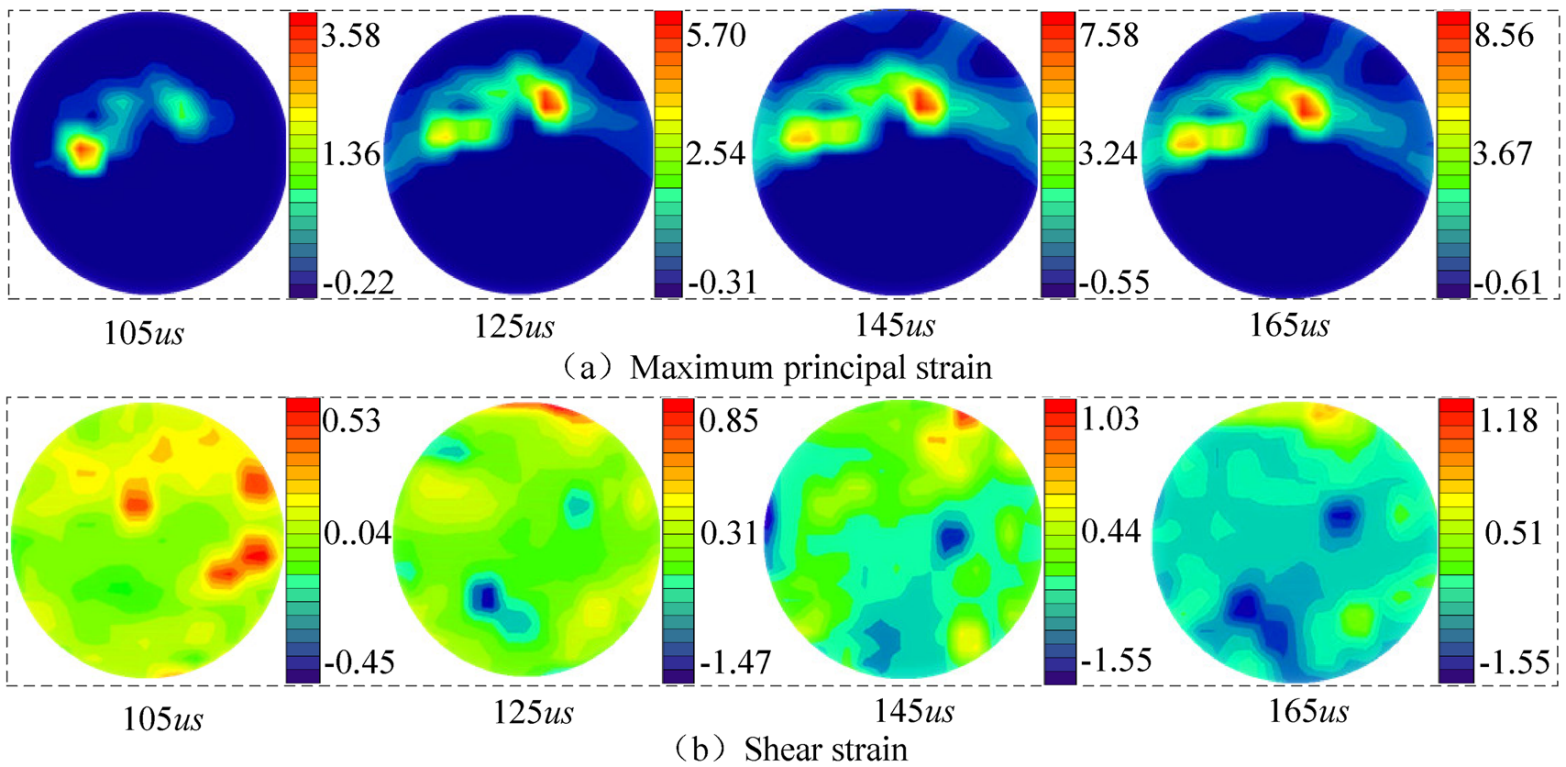
Strain field distribution in saturated coal samples at a loading pressure of 0.30 MPa.

Under the action of dynamic impact load, multiple principal strain concentration zones appear in the sample’s center, as shown in Fig. 11. The number of principal strain concentration zones is not related to the impact load magnitude. With the impact loading, the principal strain concentration zone's share increased, eventually forming a radially penetrating strain concentration area. As observed in the cloud diagram of the saturated coal sample's maximum principal strain, the strain concentration zone dimensions were different. Still, the maximum value was always located in the strain concentration zone. Several shear strain concentration zones appear in the center of the sample under the shear strain cloud diagram's impact load. However, the shear strain concentration domain has not penetrated to form an obvious strain concentration area, indicating that the samples' crack propagation had mainly a tensile form.

The crack propagation of saturated coal samples was relatively complex. Multiple principal strain concentration zones were initially formed at the central part of the coal sample. Then, under the external dynamic load, they gradually propagated and penetrated along the impact loading direction. Comparing the shear strain cloud diagram of the natural state and the saturated coal sample under the same loading air pressure, there was no particular rule in the shear strain cloud image of saturated coal samples, and the strain value is small, the shear strain value of saturated coal sample is about twice that of natural coal sample. Because the coal sample's internal water could not be rapidly eliminated due to external load and water content. Thus, high pore pressure was generated and superimposed with the impact force.

### Dynamic crack evolution

**Figure 12:**
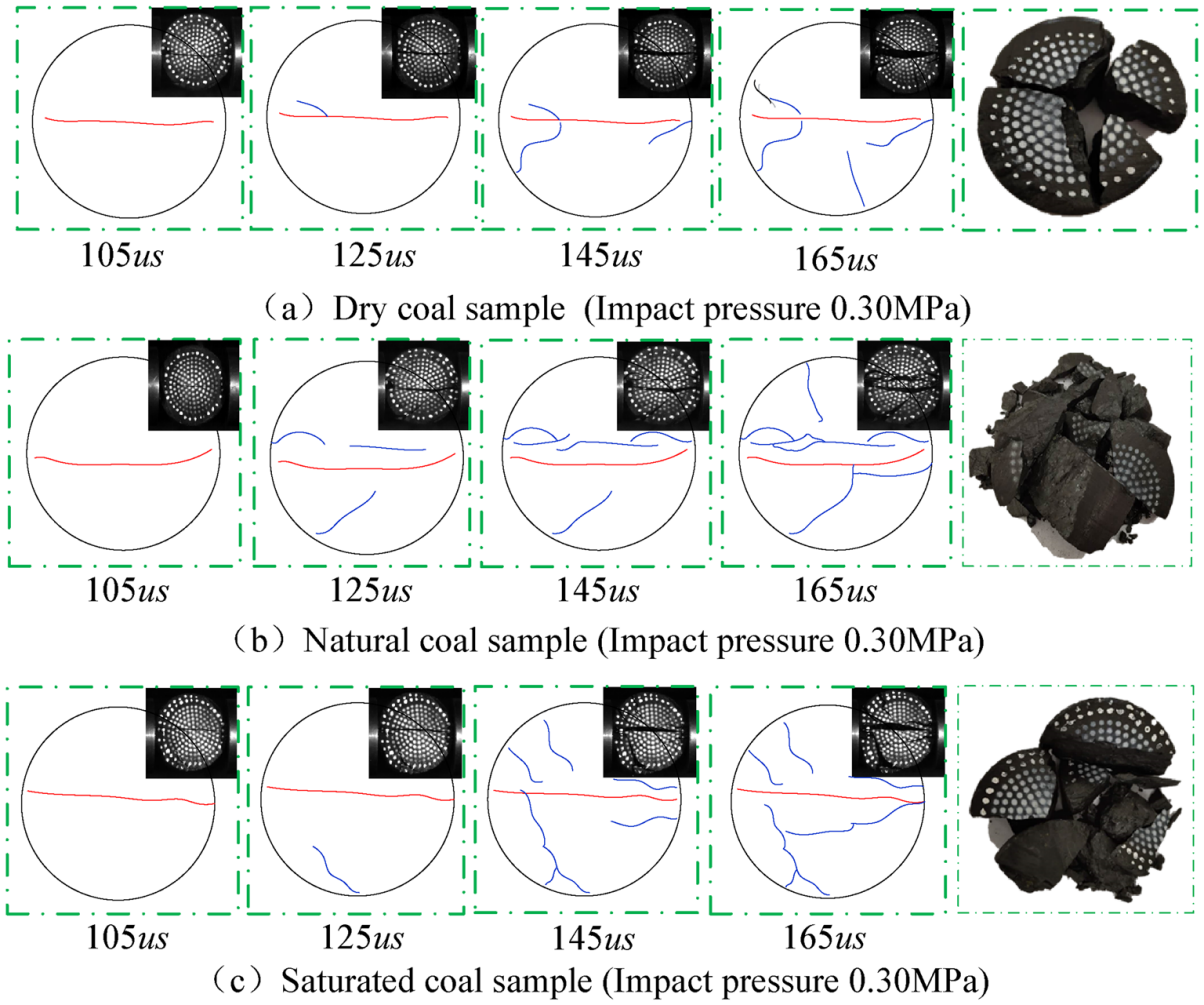
Sketching diagram of impact splitting failure process of coal samples with different water content.

The evolution features of coal sample cracks under impact load are the main research content of coal rock dynamic mechanical features^35^. The coal sample's crack propagation and failure morphology during the dynamic tensile process changes with the water content. During the SHPB test, the failure process of coal samples with different water contents is recorded by high-speed video. The surface crack evolution features were analyzed. Herein, Fig. 12 is a sketch of typical coal sample failure under impact loading. The upper right corner is a high-speed camera image of the coal sample's dynamic failure process, and the right side is the corresponding coal sample failure pattern. As observed, the degree of failure of coal samples in natural and saturated samples was higher than that of dry samples under the same loading pressure. The macroscopic crack propagation path on the surface was not smooth. Coal samples gradually decomposed and peeled under dynamic load. As shown in Fig. 12a, the number of cracks in the dry coal sample was small when it was fractured. The middle tensile crack (red line) is the main one. The failure of saturated coal samples resulted in tensile cracks along the loading direction accompanied by numerous bifurcated secondary cracks (blue line). Under external loading, secondary cracks gradually propagated to the sample edge, as shown in Fig. 12c. The coal sample was affected by the internal primary crack and water content, etc. The surface crack propagation and failure morphology exhibited different features. With an increase in water content, the number of cracks after the coal sample failure increased, and their morphology was complex. In summary, the coal sample surface crack propagation features on the coal sample surface were under dynamic impact load, and tensile cracks appeared in the center of the coal sample first. Then new cracks were generated, which propagated and finally decomposed and peeled-off in coal samples.

## Discussion

Results show that under high loading rate, water has a certain positive effect on the mechanical performances of coal samples, and its influence on the dynamic mechanical performances was mainly reflected in three aspects:

1. There are differences between mineral particles in coal samples, and clay minerals bond other mineral particles to each other, There are a large number of natural joints and fissures in the mineral itself. A series of physical and chemical reactions occur between water and minerals. Water migrates between internal micropores and fissures, which reducing the connection force between mineral particles. With the increase of water content, the internal cracks of coal samples increase, the overall integrity becomes worse, and the needle-like and sheet-like structures appear locally after saturation.
2. Generally, since the loading rate of static test is low, the inertia of coal sample can be ignored. However, in the dynamic test, the deformation and failure of coal samples occur instantaneously, and the inertia effect will prevent the generation and propagation of cracks, increase the strength of coal samples. With the increase of water content, the weight of coal samples increases, and the inertia effect of dynamic test increases.
3. The mechanical properties of water-bearing coal samples are affected by the interaction of water softening and strengthening. Under the effect of external load, the internal water of coal samples cannot be excluded, resulting in high pore pressure in the pores and fissures, which induced the expansion of stress concentration at the crack tip. When the loading rate is low, water would induced the swelling of clay mineral particles and cause the disintegration of two adjacent particles, increase the particle spacing, and then reduce the liquid bridge force. The free water at the crack tip will also promote the crack initiation and the softening effect of water is dominant when the cohesive force of the interface is small. Under higher loading rate, the inertial effect and viscous stress are enhanced, and the enhancement of water is dominant.

## Conclusions

1. The dynamic stress–strain curves of coal samples with different water content at various loading pressures contain the compaction stage, elastic (linear) deformation stage, plastic deformation stage, and failure stage. As the water content increases, the curves show a rightward shifting trend, indicating reduced brittleness, enhanced ductility, and exacerbated coal samples’ damage.
2. As the loading pressure increases, the coal sample's dynamic tensile strength is gradually improved, which has a good rate correlation. Under the same loading pressure, the coal sample's dynamic peak stress shows an asymmetric concave bending trend with increased water content. The dynamic peak stress and peak strain of coal samples with different water content versus loading pressure have a quadratic polynomial function relationship and a strong correlation.
3. The disc coal sample shows tensile splitting failure features, with the main crack propagating along the loading direction. Under the impact load, the coal sample first cracks in its center, followed by initiation, propagation, and penetration of multiple secondary microcracks, resulting in surface cracks.
4. Under dynamic loads, multiple principal strain concentration zones appear at the center of the saturated coal sample. Then, the principal strain concentration zone propagates gradually and eventually exhibits radial direction development. This phenomenon mainly occurs because it is too late to eliminate the coal sample's internal water under the external impact load, which generates high pressure in pores and fissures that becomes superimposed with the impact force.
